# Megaprepuce Reconstruction: A Single Center Experience

**DOI:** 10.3389/fped.2018.00064

**Published:** 2018-03-20

**Authors:** Miguel Luis Podestá, Miguel Podestá

**Affiliations:** ^1^Urology Unit, Department of Surgery, Hospital de Niños “R Gutiérrez”, Buenos Aires, Argentina

**Keywords:** prepuce, penis, congenital abnormalities, children, surgery

## Abstract

**Introduction:**

Surgical treatment of congenital megaprepuce is challenging and controversial. We report our 10-year experience treating patients with this deformity using a standardized procedure that has similarities to a technique reported by Smeulders et al. (1). Our postoperative complications and mid-term follow-up cosmetic appearance of the genitalia after reconstruction are reviewed.

**Material and methods:**

Fifteen patients operated on between 2005 and 2015 were evaluated. Age at surgical repair ranged from 3 to 20 months (mean 9). Treatment included unfolding the preputial sac *via* a ventral approach, excision of redundant inner preputial skin, and ventral skin coverage with the outer preputial layer. Twelve patients presented associated partial scrotal engulfment, which was simultaneously treated. Mean follow-up was 4.6 years (range 2–7 years).

**Results:**

Short-term complications occurred in three patients: scrotal hematoma in one patient and small skin dehiscence at the penoscrotal junction in two patients. Skin disruption healed by secondary epithelial ingrowth. All cases resulted in a satisfactory genital cosmetic outcome. There were no late complications. All patients preserved normal external genitalia appearance.

**Conclusion:**

Our experience is in agreement with reports of other authors; suggesting that excision of the inner preputial layer and using the external one for penile coverage provide good and durable mid-term esthetic results in megaprepuce reconstruction.

## Introduction

Megaprepuce is a rare development abnormality characterized by marked ballooning of the foreskin covering a normal-sized penis. In 1994, O’Brien et al. (2) reported this condition and named it congenital megaprepuce. Since its introduction several surgical techniques have been described for the treatment of this deformity (2–5).

Herein, we retrospectively report the outcome and mid-term follow-up in consecutive patients treated with a standardized surgical procedure excising the inner preputial layer and utilizing the outer one to cover the penile shaft. Several specific points of the technique are also briefly analyzed.

## Materials and Methods

**Figure 1:**
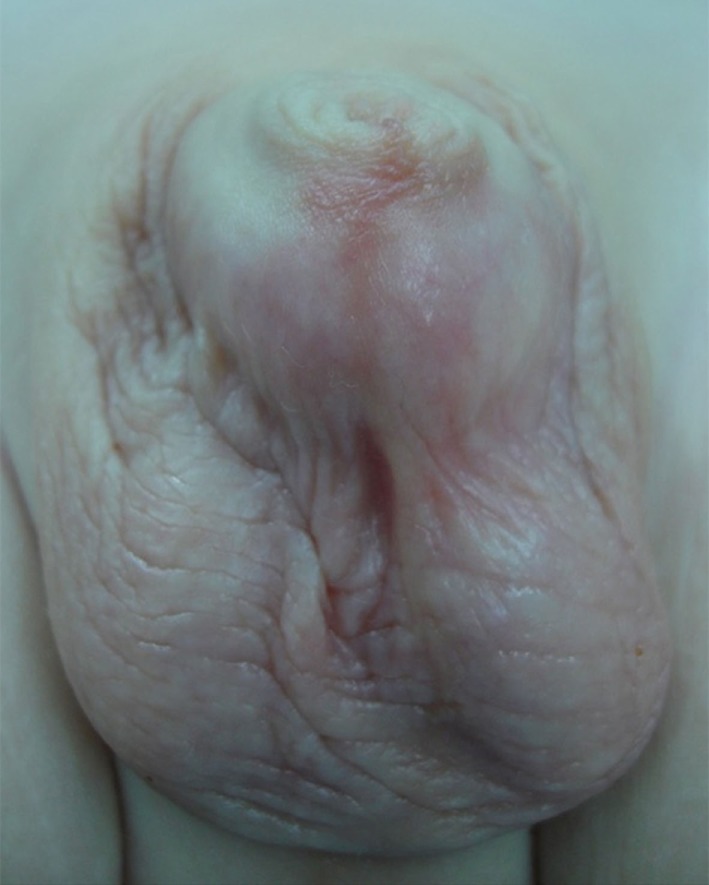
Megaprepuce in a young infant with a non-scarred preputial opening.

Between July 2005 and June 2015, 15 patients with megaprepuce, aged 3–20 months (mean 9), underwent genital reconstruction at our hospital. All cases presented with a bulging preputial sac, engulfing the penile shaft that increased during voiding (see Figure 1). Other symptoms included a history of difficulty in voiding and continual urine dribbling. On physical examination, all had a non-retractable prepuce, but without a stenotic opening, 12 presented a partial penoscrotal transposition.

**Figure 2:**
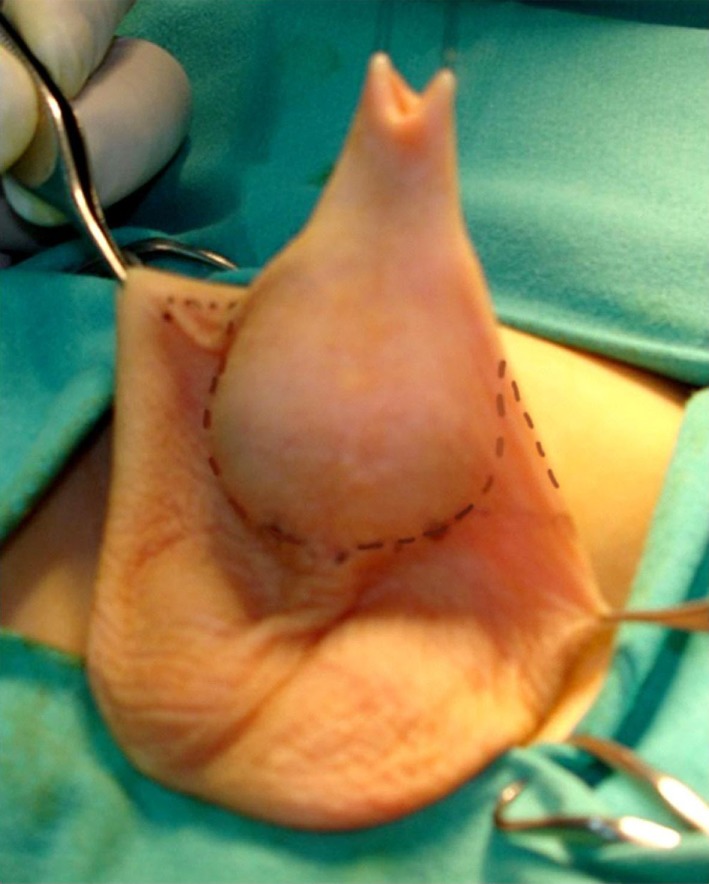
Traction in the preputial opening. Before ventral transverse incision, we marked the limit between scrotal and penile skin, which was prolonged laterally, circumscribing the apical scrotal engulfment adjacent to each side of the penile shaft.

**Figure 3:**
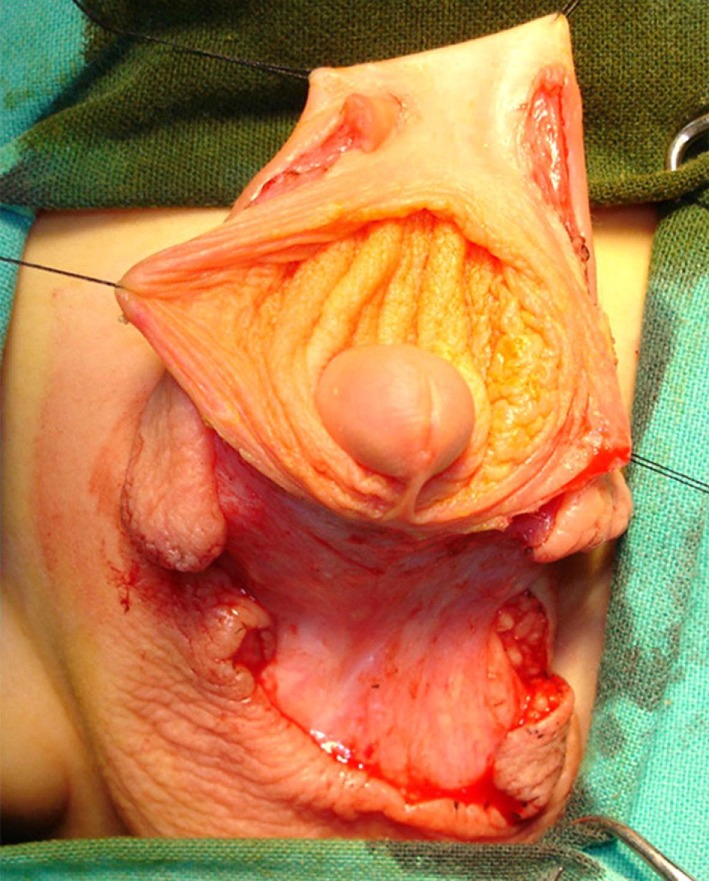
Inner preputial layer has been exposed *via* a ventral approach.

**Figure 4:**
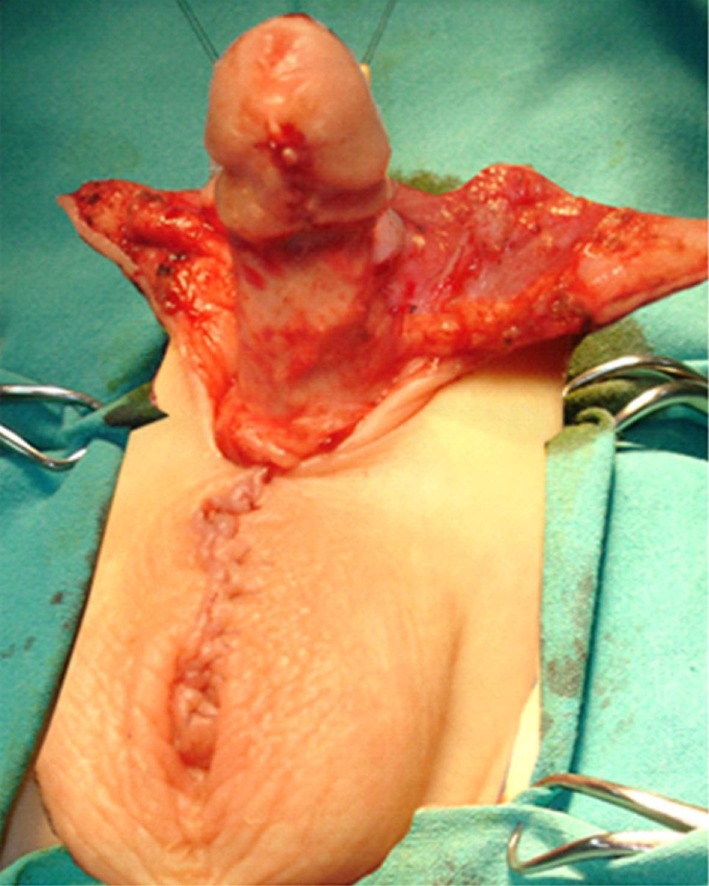
Inner foreskin layer has been excised leaving a cuff of mucosa under the glans and the outer layer for penile coverage. Scrotal flaps were dissected and sutured together in the midline.

**Figure 5:**
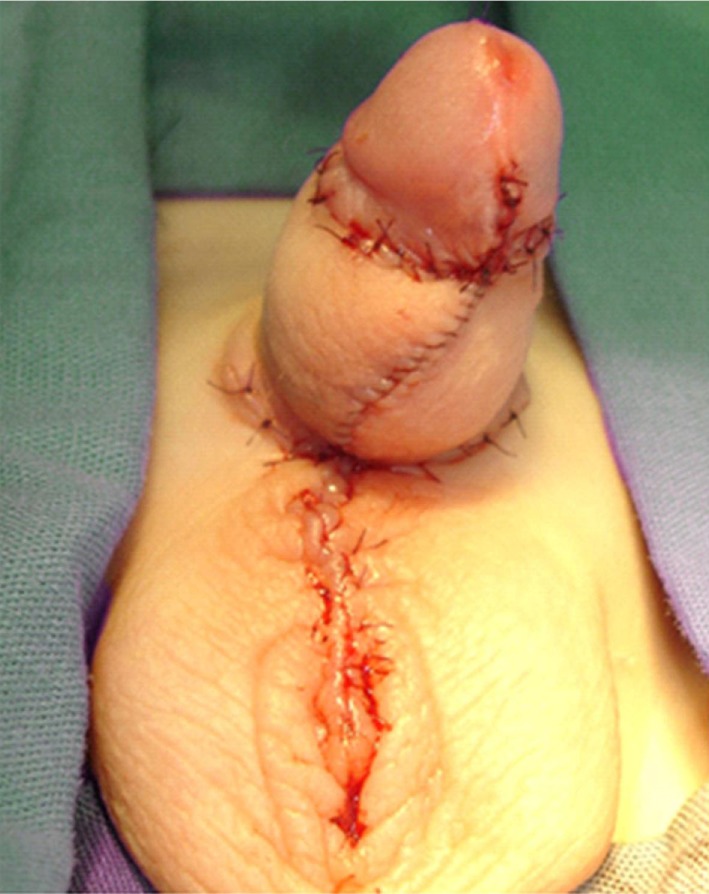
Genitalia appearance after immediate megaprepuce reconstruction.

Surgical repair is started by placing two traction sutures at the preputial opening. Ventral transverse incision was performed between the scrotal and penile skin and prolonged laterally, circumscribing the apical scrotal engulfment adjacent to each side of the penile shaft (Figure 2). Scrotal flaps were dissected and rotated medially beneath the penis and sutured together in the midline. The preputial sac was opened, and the inner layer exposed using a longitudinal ventral incision (see Figure 3). Redundant inner layer was dissected from the penile shaft, carefully separated from the outer preputial layer and excised, leaving a cuff of mucosa attached to the glans (Figure 4). When dysgenic tissue bands are identified; they were removed from the albuginea of the corpora cavernosa. Next, the penile shaft was covered with dorsal preputial and penile skin, in a Byars’ fashion. Finally, the skin around the penis was approximated to the distal mucosal cuff and to the scrotal skin (Figure 5) Urinary diversion with a silicone catheter was left for 3 days, draining in a double-diaper system. A bioocclusive dressing (Tegaderm) was maintained around the penis for 5 days. Intraoperative caudal anesthetic block was routinely performed. This surgical procedure has similarities with other methods previously used in the treatment of this abnormality (1, 4, 5). Follow-up ranged from 2 to 7 years (mean 4.6).

### Ethical Approval

Approval was granted by the hospital ethics review board.

## Results

**Figure 6:**
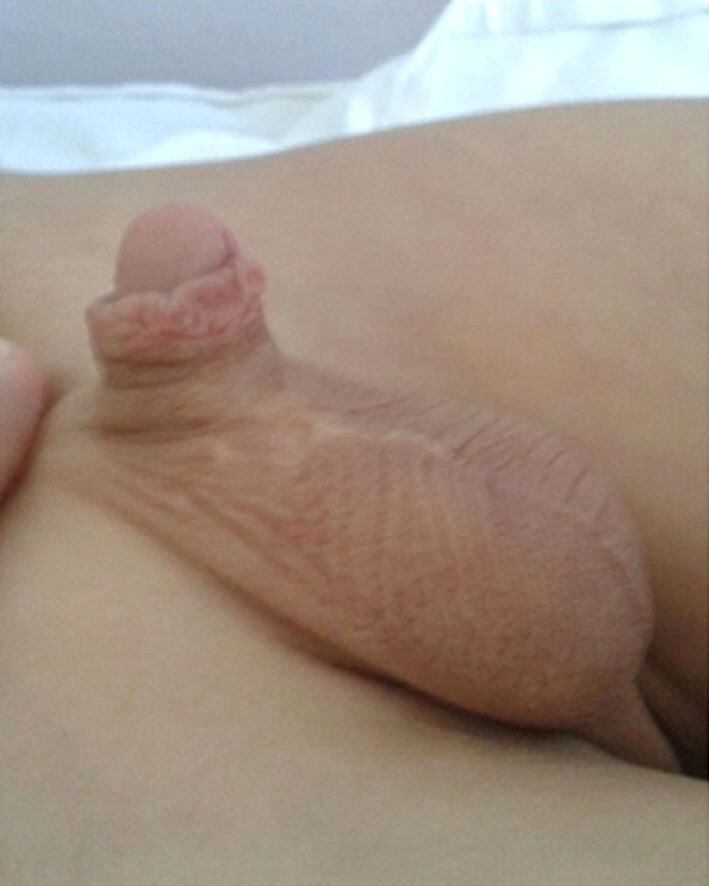
The same patient 4 years later.

In all cases, surgical reconstruction showed an excessive inner foreskin layer that went down to the base of the penis and returned to the coronal sulcus, causing a big preputial pouch. Complications after surgery included a scrotal hematoma in one patient and in two cases skin dehiscence at the penoscrotal junction. These last two complications healed by secondary epithelial ingrowth. Postoperatively, all patients recovered a normal micturition pattern and achieved a nice genital appearance. At median follow-up, genitalia remained with a normal esthetic appearance without the need of further surgical procedures (Figure 6).

## Discussion

Megaprepuce is a very uncommon deformity in which a normal phallus is covered by dilated foreskin. Debate continues on whether this condition is congenital or acquired. Some authors proposed the occurrence of megaprepuce as a result of an in-folding of the prepuce causing severe phimosis (6, 7). However, O’Brien et al. (2) suggested that although these patients present non-retractable foreskin, there is no evidence of true phimosis. Later on Philip and Nicholas (8) reported that this anomaly was the consequence of an abnormal attachment of the inner layer of the prepuce to the coronal sulcus and not secondary to phimosis. A further explanation of the etiology of this deformity is attributed to a detachment failure of the tissue planes migration in the developing male external genitalia (1).

In this retrospective study, all cases were diagnosed in early infancy and consistently had a narrow preputial orifice though not stenotic or scarred, associated with a redundant inner layer of the prepuce. We speculate that this condition is congenital.

Several surgical methods have been proposed for treating this condition. Some of them are based on the management of the concealed penis, which is a deformity anatomically different to the congenital megaprepuce. In the present cohort of patients, treatment was directed toward unfolding the preputial sac *via* a ventral approach, excising the excessive inner preputial skin and using the outer layer for penile coverage, following Smeulders et al. technique (1).

Partial scrotal transposition was associated with the megaprepuce in 80% of the cases. Thus, we elected to extend the ventral skin incision at the penoscrotal junction, surrounding the partial scrotal engulfment on either side of the penile shaft, following Leao et al. technique (5).

In contrast to surgical procedures in which the preputial sac is unfurled, and the inner layer is preserved to resurface the penile shaft, we have routinely excised the excessive inner layer and used the outer one to cover the penis. In addition, when dysgenetic tissue bands on the ventral side of the penis were present, they were resected to the level of the tunica albuginea of the corporeal bodies (9).

With the reconstructive procedure described herein, two patients developed a small ventral midline skin dehiscence at the penoscrotal junction. Causes for these small breakdowns of preputial skin may be related to poor venous backflow with risk of partial necrosis or to excessive skin tension at the midline closure. In all patients, the external genitalia had a sustained normal appearance at the end of follow-up. No further surgical procedures was performed in their genitalia.

Usually, techniques that preserve the inner layer to cover the penile shaft have led to poor esthetic results, needing more redo surgery to correct the abnormal appearance of the penile skin. In their earlier cases, Summerton et al. (3) excised the outer preputial skin and used the inner layer to resurface the penile shaft. Failure to obtain adequate cosmetic results made them modify the technique, utilizing the outer layer for penile coverage and resecting the internal layer.

In 26 patients with megaprepuce, Ruiz et al. (10) performed an initial dorsal slit or a “limited” circumcision using the inner preputial skin to resurface the penis, based on the technique described by Donahue and Keating (11) to correct the buried penis. Postoperatively, their cases developed penile skin redundancy, though none required reoperation. Later on, Rod et al. (12) treated 52 patients with megaprepuce preserving and trimming the inner preputial layer with very good postoperative results in only 44% of their cases and acceptable ones in 46% with a reoperation rate of 8%. Again these authors performed a circumferential incision beneath the preputial opening. Furthermore, Ferro et al. treated six patients with complete exteriorization of the shaft, section of the penile ligament, and restoration of the pubo-penis and penoscrotal angles. For penile coverage, they used the shaft penile skin and part of the inner layer, transposed ventrally (13). With a rather similar procedure on how to resurface the penis, Alexander et al. treated 10 patients with megaprepuce (14). These authors divided the stenotic preputial ring and used a triangular flap of tissue from the inner foreskin to achieve adequate skin coverage. These two studies have a very short follow-up of their patients. The surgical procedure presented in our study has the advantage “to diminish” the edema and often redundancy of the inner preputial layer by using the dorsal layer to cover the penis and excise completely the inner one as well as the dysgenetic bands on the ventral surface of the albuginea, if present. This surgical procedure shows good esthetic results with a mean follow-up of 4.6 years.

It should be noted that Murakami et al. (15) reported 96.9% good postoperative penile cosmetic results in a larger series of patients (65 cases) treated with a technique similar to the one described in this study. Only 3.1% of their cases needed redundant penile skin excision.

In agreement with other authors, we do not recommend initial circumcision as part of megaprepuce reconstruction, as this surgical step may remove skin needed for penile coverage (1, 5, 16).

A limitation of our study is the method used for assessing surgical results, which was based on the surgeon’s opinion, rather than on a standardized validated questionnaire filled by parents.

In summary, the technique described herein has provided nice and durable penile appearance with an initial low complication rate. Our data suggest that excision of the inner foreskin layer and penile coverage with the dorsal one provides better cosmetic results than with the use of the inner layer to resurface the penis and confirms the reports of other authors (1, 5, 15).

## Conclusion

Although the number of patients in this study was small, the mid-term follow-up of this technique showed good and durable esthetic results of the genitalia with minimal postoperative complications. We recommend in accordance with the experience of others, excising the inner preputial layer and using the dorsal one for penile coverage.

## Ethics Statement

This retrospective review was approved by the ethical committee of the Hospital de Niños Ricardo Gutierrez from Buenos Aires Argentina.

## Author Contributions

Both authors (MLP and MP) contributed equally to the design and writing to this article.

## Conflict of Interest Statement

The authors declare that the research was conducted in the absence of any commercial or financial relationships that could be construed as a potential conflict of interest.
